# A study of machine learning to predict NRDS severity based on lung ultrasound score and clinical indicators

**DOI:** 10.3389/fmed.2024.1481830

**Published:** 2024-11-01

**Authors:** Chunyan Huang, Xiaoming Ha, Yanfang Cui, Hongxia Zhang

**Affiliations:** ^1^Department of Ultrasound, Yantaishan Hospital, Yantai, China; ^2^Medical Impact and Nuclear Medicine Program, Binzhou Medical University, Yantai, China

**Keywords:** NRDS, machine learning, risk factor, clinical indicator, prediction model

## Abstract

**Objective:**

To develop predictive models for neonatal respiratory distress syndrome (NRDS) using machine learning algorithms to improve the accuracy of severity predictions.

**Methods:**

This double-blind cohort study included 230 neonates admitted to the neonatal intensive care unit (NICU) of Yantaishan Hospital between December 2020 and June 2023. Of these, 119 neonates were diagnosed with NRDS and placed in the NRDS group, while 111 neonates with other conditions formed the non-NRDS (N-NRDS) group. All neonates underwent lung ultrasound and various clinical assessments, with data collected on the oxygenation index (OI), sequential organ failure assessment (SOFA), respiratory index (RI), and lung ultrasound score (LUS). An independent sample test was used to compare the groups’ LUS, OI, RI, SOFA scores, and clinical data. Use Least Absolute Shrinkage and Selection Operator (LASSO) regression to identify predictor variables, and construct a model for predicting NRDS severity using logistic regression (LR), random forest (RF), artificial neural network (NN), and support vector machine (SVM) algorithms. The importance of predictive variables and performance metrics was evaluated for each model.

**Results:**

The NRDS group showed significantly higher LUS, SOFA, and RI scores and lower OI values than the N-NRDS group (*p* < 0.01). LUS, SOFA, and RI scores were significantly higher in the severe NRDS group compared to the mild and moderate groups, while OI was markedly lower (*p* < 0.01). LUS, OI, RI, and SOFA scores were the most impactful variables for the predictive efficacy of the models. The RF model performed best of the four models, with an AUC of 0.894, accuracy of 0.808, and sensitivity of 0.706. In contrast, the LR, NN, and SVM models have lower AUC values than the RF model with 0.841, 0.828, and 0.726, respectively.

**Conclusion:**

Four predictive models based on machine learning can accurately assess the severity of NRDS. Among them, the RF model exhibits the best predictive performance, offering more effective support for the treatment and care of neonates.

## Introduction

Neonatal respiratory distress syndrome (NRDS) significantly contributes to neonatal mortality, primarily due to a deficiency of pulmonary surfactant, a critical factor in its complex pathogenesis (1). Timely diagnosis and intervention are crucial for improving the prognosis of affected neonates. Currently, the primary methods for diagnosing NRDS include clinical symptom assessment, imaging studies, and blood gas analysis to monitor respiratory and multi-organ function (2, 3). OI and RI are critical indicators of pulmonary function, widely used to assess the severity of respiratory distress in neonates, and are strongly associated with pulmonary developmental abnormalities (4, 5). These metrics objectively reflect the oxygenation status of neonates and are vital tools for assessing pulmonary ventilation and gas exchange.

The SOFA score is a clinical tool used to evaluate the degree of multi-organ dysfunction in pediatric patients, widely applied in intensive care units and for assessing critically ill neonates. In clinical practice, the severity of NRDS is typically assessed using the OI, RI, and SOFA scoring systems (6). Pulmonary ultrasound effectively reflects pulmonary ventilation, and LUS is well-documented for quantifying ventilation status, particularly in adults (7). Several scoring systems are available for quantitatively assessing pulmonary ultrasound in adults. Studies indicate that LUS scoring in pulmonary ultrasound examinations effectively reflects NRDS severity (8). In some regional hospital ICUs, pulmonary ultrasound has almost replaced traditional chest radiography (9). However, some studies suggest that due to various factors influencing pulmonary ultrasound results, traditional chest radiographs may be more effective for diagnosing NRDS. The clinical applications of quantitative ultrasound methods for diagnosing pulmonary diseases and assessing their severity are now widespread.

Recent years have shown significant potential for applying artificial intelligence in the medical field (10, 11). Machine learning, a vital tool for data mining, offers greater flexibility and scalability than traditional statistical methods, effectively handling multivariable interactions and collinearity (12). Machine learning uses existing medical testing or patient survey data to establish risk models, enabling disease prediction, diagnosis, and severity assessment (13). Research shows that machine learning algorithms in AI can develop efficient diagnostic and predictive tools, improving tumor diagnosis accuracy by 15 to 20% (14–16). In many prospective studies, machine learning models perform better than medical experts (17, 18). This could be due to the reduced human intervention in AI, minimizing biases and subjective errors in predictions.

This study aims to develop four machine learning models to predict NRDS severity based on clinical indicators and LUS, identifying the most effective models to support NRDS treatment. It will enhance understanding of the relationship between pulmonary pathology, respiratory function, and systemic organ function in neonates with NRDS. This will provide clinicians with a more precise basis for assessing affected neonates and offer a scientific foundation for diagnosing and treating respiratory diseases.

## Methods

### Experimental design

This double-masked cohort study was conducted in the NICU to assess NRDS severity in neonates using LUS and other clinical indicators. The entire research process was conducted within the NICU to ensure legality and ethical integrity. NICU clinical management adhered to local guidelines, with the study not directly influencing clinical practices. Researchers ensured neonates received appropriate treatment and care while collecting clinical data and LUS scores for subsequent analysis. Researchers used the STROBE checklist during manuscript preparation to ensure the study’s reliability and scientific rigor.

### Patients

This study involved 336 pediatric patients admitted to the NICU at Yantaishan Hospital from December 2020 to July 2023. Neonates were selected based on strict inclusion criteria to ensure they met the diagnostic standards for NRDS. From the original cohort, 106 patients were excluded for reasons including an unclear diagnosis, withdrawal from treatment, congenital anomalies, tuberculosis, or congestive heart failure. As a result, 230 neonates remained in the study, with 119 identified as having NRDS and 111 categorized as N-NRDS. The diagnostic procedures were carried out using a Philips CX50 portable ultrasound machine equipped with a linear array probe, operating at a frequency range of 8 to 12 MHz. The study protocol adhered to medical ethical standards and received approval from the Medical Ethics Committee of Yantai Mountain Hospital (approval number: 20220001), with informed consent obtained from the patients’ families. All data collection and processing followed relevant guidelines and standards.

### Inclusion criteria and exclusion criteria

The inclusion criteria for this study were grounded in the Berlin criteria, encompassing clinical symptoms, arterial blood gas analysis, and chest X-ray results. A diagnosis of NRDS was confirmed by evaluating clinical signs, arterial blood gas measurements, and radiographic evidence. Eligible neonates were those born within 6 to 12 h before disease onset, showing severe respiratory distress, cyanosis, expiratory grunting, inspiratory retractions, and related symptoms. Arterial blood gas analysis required evidence of hypoxemia, defined as an arterial oxygen partial pressure below 60 mmHg, or hypercapnia, indicated by a carbon dioxide partial pressure exceeding 50 mmHg. Chest X-rays showed decreased lung transparency, increased lung markings, evenly distributed granulation and reticular opacities, blurred cardiac and diaphragmatic outlines, bronchial aeration, and signs of severe pneumonia in both lung fields. Inclusion required that the neonate have a complete clinical medical record.

The exclusion criteria for this study include neonates with congenital disorders such as complex respiratory malformations, congenital heart disease, congenital mental disorders, or chromosomal abnormalities. Neonates showing signs of cardiogenic pulmonary edema or persistent pulmonary hypertension. Neonates with severe intracranial hemorrhage, sepsis, severe hypoxic–ischemic encephalopathy, severe sepsis, septic shock, or other serious illnesses and multi-system complications. Neonates diagnosed with pulmonary hemorrhage, neonatal wet lung, pneumothorax, meconium aspiration syndrome, neonatal infectious pneumonia, or other respiratory disorders. Additionally, neonates were excluded if factors led to increased measurement errors or significantly compromised ultrasound image quality.

### Observation and evaluation indicator

**Figure 1:**
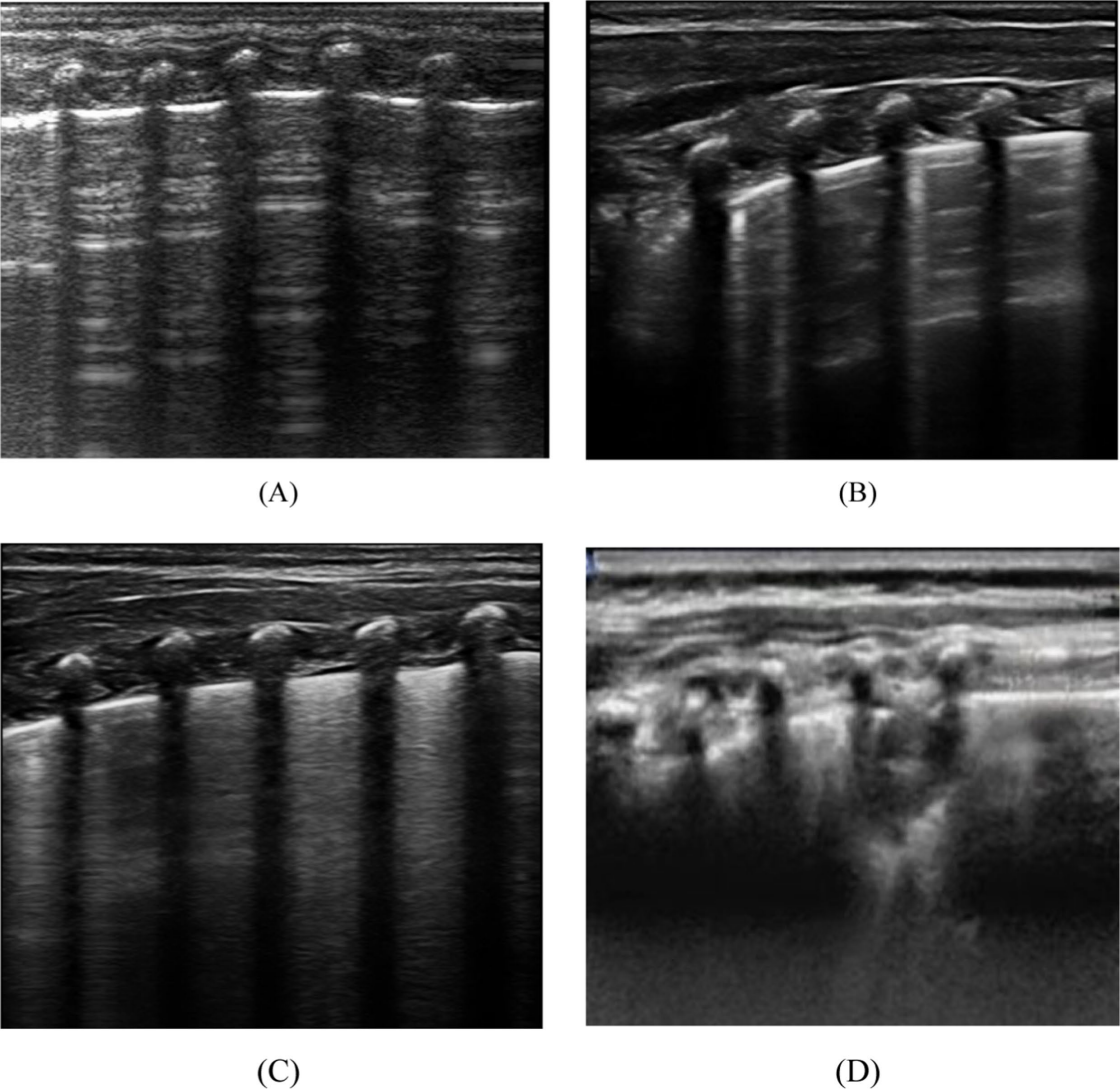
Depicts the four distinct lung ultrasound scoring patterns. **(A)** A score of 0 is assigned when only A lines are visible or fewer than 3 B lines. A vertical arrow indicates A-lines. **(B)** A score of 1 is given when there are 3 or more well-spaced B-lines, shown with horizontal arrows. **(C)** Score 2 was line B with many fusions, with or without subpleural consolidation. **(D)** 3 points are defined as “hepatoid” lung tissue or accompanied by an air bronchogram, represented by an oval.

Lung ultrasound score has diverse applications in clinical assessment (19). This study employed a 12-region scoring system, dividing the pediatric chest into 12 zones, with each lung region systematically scanned and scored based on specific ultrasound images, leading to the calculation of the LUS score. The scoring criteria outlined in Table 1 provide a standardized approach for the quantitative evaluation of pulmonary diseases and their severity. Continuous, smooth A-lines or the presence of fewer than three isolated B-lines are assigned a score of 0, indicating normal lung status. A score of 1 is given when three or more scattered, distinct B-lines reflect a mild abnormality. A score of 2 is assigned for diffusely confluent B-lines appearing in a waterfall pattern, indicating moderate abnormality. Lung consolidation, with a score of 3, indicates severe abnormality. The total LUS score ranges from 0 to 36, as depicted in Figure 1, which presents representative images from our institution’s assessment of NRDS scores between 0 and 3 (20).

**Table 1:** LUS evaluation criteria (20).

	Score
Lung consolidation	3
Numerous integrated B-lines (waterfall sign)	2
Scattered on clear B-lines (≥3)	1
Smooth A-lines or fewer than 3 isolated B-lines	0

The OI is a critical measure for assessing the severity of NRDS, calculated as the ratio of arterial oxygen partial pressure (PaO_2_) to the fraction of inspired oxygen (FiO_2_) (21). The formula for its calculation is OI = PaO_2_/FiO_2_. Typically, the normal range for the oxygenation index is between 400 and 500 mm Hg; lower scores suggest worsening pulmonary function and oxygenation capacity in the neonate, thereby indicating the severity of the condition. RI is crucial for assessing children’s respiratory function. It is calculated as the ratio of the alveolar-arterial oxygen pressure difference (A-aDO_2_) to PaO_2_. The formula for its calculation is RI = A-aDO_2_/PaO_2_. RI between 0.8 and 1.2 is considered normal, indicating good respiratory function, regular lung activity, and sufficient gas exchange. An RI between 1.2 and 1.6 suggests mild abnormalities, potentially indicating mild dyspnea due to respiratory muscle fatigue or upper respiratory tract infection. An RI of 1.6 to 2.0 indicates moderate abnormalities, usually signifying moderate dyspnea and pulmonary function decline, possibly due to conditions like chronic obstructive pulmonary disease. An RI exceeding 2.0, particularly above 3.0, indicates severe abnormalities, often reflecting significant respiratory dysfunction, potentially due to severe pulmonary diseases or respiratory acidosis, leading to substantial dyspnea and critically impaired lung function.

The SOFA score includes six systems: respiratory, coagulation, liver, circulatory, central nervous, and renal (22). Each system is scored from 0 to 4, with the total SOFA score being the sum of these scores. Table 2 shows that when evaluating the SOFA score in children with NRDS, special attention should be given to each system’s score, as a higher total score indicates more severe organ dysfunction. According to the criteria, the SOFA score typically ranges from 0 to 24.

**Table 2:** SOFA score table.

System	Evaluation index	Score
Coagulation system		
	>150	0
	100 ~ 150	1
	50 ~ 100	2
	20 ~ 50	3
	<20	4
Central nervous system		
	<110	0
	110 ~ 170	1
	171 ~ 299	2
	300 ~ 440	3
	>440	4
Liver		
	<20	0
	20 ~ 32	1
	33 ~ 101	2
	102 ~ 204	3
	>204	4
Respiratory system		
	>400 mmHg	0
	300 ~ 400 mmHg	1
	200 ~ 300 mmHg	2
	100 ~ 200 mmHg	3
Kidney function		
	<110	0
	110 ~ 170	1
	171 ~ 299	2
	300 ~ 440	3
	>440	4
Cardiovascular system		
	Map ≥ 70 mmHg	0
	Map < 70 mmHg	1
	Dopamine ≤5 or dobutamine dose	2
	Dopamine >5 or norepinephrine ≤0.1	3
	Dopamine >15 or norepinephrine >0.1	4

### Machine learning model

**Figure 2:**
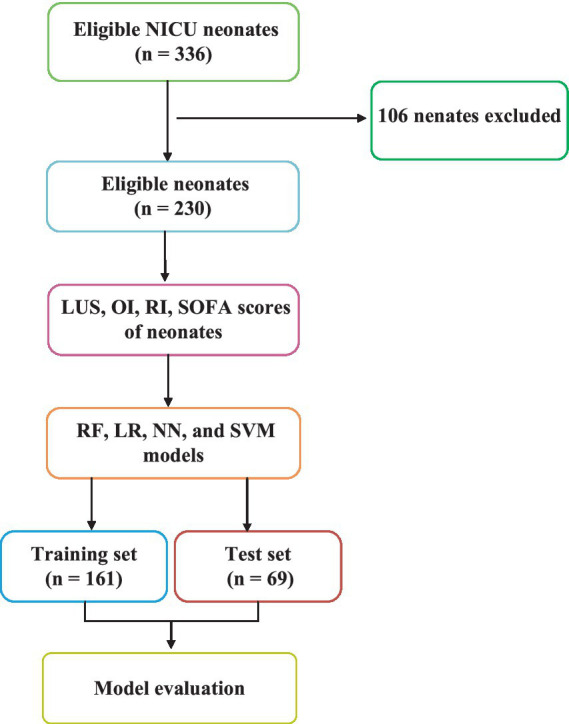
Flow diagram of the study design for neonates.

Figure 2 shows the flowchart of this study. This study addresses a binary classification problem ideally suited for supervised learning in machine learning. Supervised learning techniques encompass a variety of algorithms such as linear and LR, SVM, Naive Bayes, extreme gradient boosting, decision trees, and RF. In this research, we selected LR, RF, NN, and SVM as the training models commonly used in medical treatment.

### Machine learning model building process

Data sets with statistically significant differences were selected to construct machine learning metrics. Subsequently, 161 cases (70%) were randomly chosen from the entire sample as the training set. The remaining 69 cases (30%) were used as the test set. The training set data underwent five-fold cross-validation to achieve the optimal training model. The advantage of cross-validation is that it uses all data for both training and validation sets, with each subset independently validated, providing a more robust reflection of the training model. Finally, an independent external validation set was selected, consisting of 44 NRDS and 43 N-NRDS patients, to test and evaluate the model’s accuracy.

### Statistical analysis

Data entry was performed using SPSS software. Continuous variables that followed a normal or approximately normal distribution were expressed as mean ± standard deviation ( $\bar{x}$ ±s), with group comparisons made using the independent samples t-test. For continuous variables not following a normal distribution, data were reported as median (interquartile range), and group differences were assessed using non-parametric rank-sum tests. All data analyses and the construction of machine learning models were performed using R software (version 4.0.3). All models were built using the Caret package, with the importance of predictive variables assessed through the vamp function within Caret. In this study, a *p-*value of <0.05 was considered statistically significant.

## Results

### Comparison of primary clinical data

Table 3 presents the baseline characteristics of the two groups. In the NRDS cohort, 47.90% of the participants were male, and the median gestational age was 32 (27,36) weeks. The mean birth weight was 2013 ± 622 g, and 60.50% of deliveries were by cesarean section. In the N-NRDS group, the proportion of males was 60.30%, the median gestational age was 35 (30,39) weeks, the mean birth weight was 2,291 ± 741 g, and cesarean deliveries accounted for 54.05% of the total deliveries.

**Table 3:** Characteristic parameters of patients.

	NRDS group *n* = 119	N-NRDS group *n* = 111	*P-*value
Sex (%)			
Male	57 (47.90%)	67 (60.30%)	0.131
Cesarean section (%)	72 (60.50%)	60 (54.05%)	<0.01
Weight (g)	2013 ± 622	2,291 ± 741	<0.01
Gestational age (range)	32 (27,36)	35 (30,39)	<0.01
<Number of children at 28 weeks	76 (63.87%)	73 (65.77%)	0.107
<The Number of children with 1,500 grams	51 (42.86%)	40 (36.04%)	0.013
Apgar score			
1 min (≤7)	74 (62.18%)	55 (49.55)	0.610
5 min (≤7)	48 (40.33%)	26 (18.92%)	0.861

Values are expressed as mean ± standard deviation, median (25th percentile; 75th percentile), or Number (%); the difference between groups was statistically significant (*p* < 0.05).

### Comparison of clinical indicators among different cohorts

The LUS and clinical score variations across different patient groups were compared, as shown in Table 4. The NRDS group exhibited higher LUS, SOFA, and RI values than the N-NRDS group, while OI values were lower (*p* < 0.01).

**Table 4:** LUS and clinical scores of children in different cohorts.

	Total *n* = 330	NRDS group *n* = 119	N-NRDS group *n* = 111	*t/Z*	*P*
LUS	21.33 ± 7.12	21.88 ± 6.87	20.51 ± 7.71	−0.33	<0.01
OI	181.7 ± 75.7	179.2 ± 75.3	183.5 ± 76.3	1.91	<0.01
SOFA	16.68 ± 4.21	17.3 ± 4.83	16.2 ± 3.81	0.97	<0.01
RI	1.51 ± 0.52	1.65 ± 0.56	1.29 ± 0.49	−0.59	<0.01

NRDS is neonatal respiratory distress syndrome, LUS is lung ultrasound score, OI is oxygenation index, RI is respiratory index, and SOFA is sequential organ failure assessment.

### Comparison of different severity of NRDS patients

Patients with NRDS were categorized based on the severity of their pulmonary disease into mild, moderate, and severe groups. Table 5 displays LUS levels and clinical scores corresponding to various degrees of NRDS, revealing significant statistical differences among the groups (*p* < 0.01). The severe NRDS group showed significantly elevated LUS, SOFA, and RI scores relative to the mild and moderate groups, whereas the OI was notably lower.

**Table 5:** Comparison of LUS and clinical index scores of NRDS patients with different severity.

		LUS	OI	SOFA	RI
Mild	38	14.68 ± 6.73	233.1 ± 83.6	10.79 ± 4.01	1.19 ± 0.79
Moderate	48	20.01 ± 5.47^a^	161.1 ± 69.1^a^	15.01 ± 4.83^a^	1.55 ± 0.56^a^
Serious	33	27.12 ± 7.1^b^	111.7 ± 57.8^b^	19.36 ± 5.11^b^	2.21 ± 1.12^b^
*F*		11.22	17.32	9.76	4.53
*P*		<0.01	<0.01	<0.01	<0.01

Compared with the mild group, ^a^*P* < 0.01. Compared with the moderate group, ^b^*P* < 0.01.

### Model training process

To develop the NRDS predictive model, four commonly used machine learning algorithms were employed: LR, RF, NN, and SVM. Based on this foundation, ensemble and cascading methods were used to combine the individual classification models, and the performance of the integrated classifier was subsequently evaluated.

### Importance of predictors of different models

**Figure 3:**
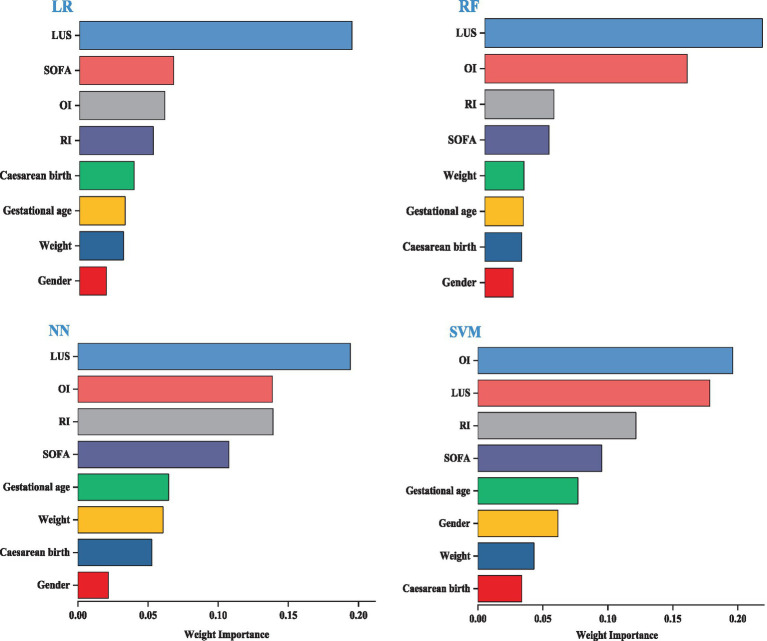
Importance of predictors for different models.

We visualized the top eight variables according to their weights. The length of the bars is directly proportional to the importance of each variable. The results indicate that the model’s key variables fall into two groups, with the first group—LUS, OI, SOFA, and RI—significantly impacting predictive performance. LUS is the most influential variable in the LR, RF, and NN models. LUS is the most influential variable for the LR, RF, and NN models. The second group consists of cesarean birth, gestational age, weight, and gender, which have a relatively minor impact on prediction; detailed information is provided in Figure 3.

### Evaluation results of different machine learning algorithms

**Figure 4:**
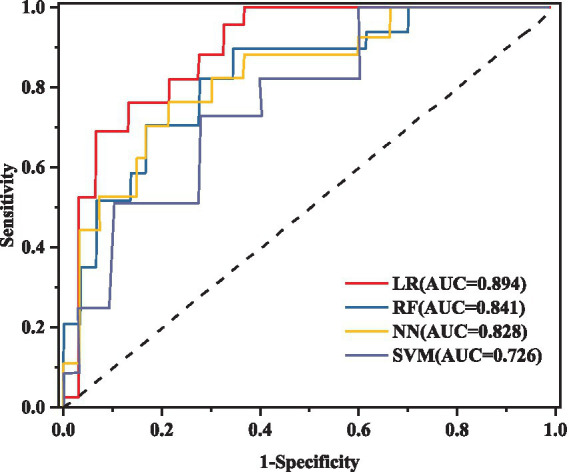
ROC curve analysis of different models in the test set.

This research utilizes five key performance metrics, the AUC, accuracy, sensitivity, specificity, and the F1 score, to assess the effectiveness of four machine learning algorithms, as detailed in Table 6. ROC curves were plotted using models constructed from the training dataset (Figure 4), with test set AUC values as follows: LR (AUC = 0.841, 95% CI: 0.712–0.881), RF (AUC = 0.894, 95% CI: 0.811–0.976), NN (AUC = 0.828, 95% CI: 0.701–0.935), and SVM (AUC = 0.726, 95% CI: 0.623–0.887). The RF model demonstrated the highest performance of the four models, with an AUC of 0.894, accuracy of 0.808, and sensitivity of 0.706. In contrast, the AUC values for the LR, NN, and SVM models were notably lower. Given the superior performance of the RF model, it was selected as the final model for this study.

**Table 6:** The performance of different machine learning algorithm models.

	AUC	Accuracy	Sensitivity	Specificity	F1
LR					
Train	0.870	0.818	0.691	0.861	0.727
Test	0.841	0.771	0.613	0.877	0.661
RF					
Train	0.872	0.801	0.679	0.871	0.704
Test	0.894	0.808	0.706	0.867	0.727
NN					
Train	0.847	0.778	0.632	0.733	0.681
Test	0.828	0.786	0.593	0.851	0.653
SVM					
Train	0.670	0.731	0.754	0.776	0.693
Test	0.726	0.766	0.704	0.801	0.686

### Verification of the model

**Figure 5:**
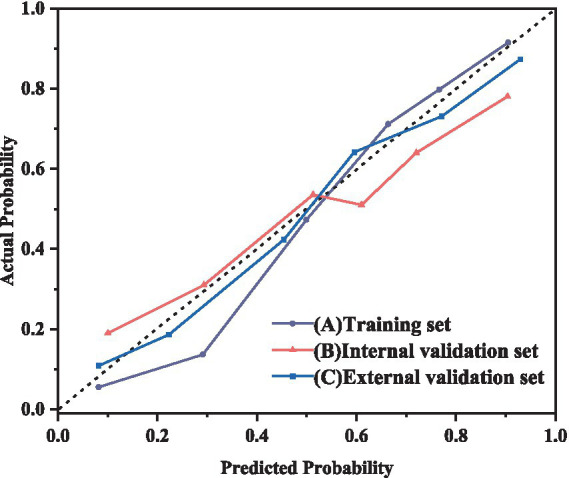
Calibration curve of RF model for predicting the severity of NRDS patients A: training set; B: internal validation set; C: external validation set.

This study used calibration curves to evaluate the accuracy of the model. Figure 5 depicts the calibration curves for the training, internal validation, and external validation sets. The results show that the nomogram’s predictive performance across all sets is closely aligned with ideal performance.

## Discussion

In recent years, NRDS has garnered widespread attention. NRDS manifests as progressive respiratory distress and respiratory failure shortly after birth (23). Previous studies, both domestic and international, have shown that preterm neonates are at significantly higher risk of developing NRDS, posing a severe threat to neonatal health (24). For example, preterm neonates born at 28–29 weeks have an 81% probability of developing NRDS (25). Similar studies in Europe have yielded consistent results, showing an 80% incidence of NRDS among preterm neonates born before 28 weeks. Without timely intervention, NRDS can lead to bronchopulmonary dysplasia and even pose a life-threatening risk to the neonatal (26, 27). Therefore, early identification and timely intervention in neonates with NRDS are crucial for improving survival rates and outcomes.

Among clinical indicators, OI, SOFA, and RI are crucial for assessing NRDS in patients (28). OI assesses neonates’ respiratory function and oxygenation levels, making it a key parameter for determining NRDS severity and predicting outcomes (29). Monitoring OI during NRDS treatment helps assess the neonate’s oxygenation status promptly, enabling adjustments in treatment to enhance respiratory support and improve oxygenation. SOFA is a valuable clinical tool for evaluating the severity of multiple organ dysfunction in neonates with NRDS (30). SOFA scores various organs to assess disease severity and prognosis, offering crucial guidance for clinical decision-making. RI is another essential indicator for NRDS, calculated using gas exchange principles to reflect the lung’s ability to diffuse oxygen and exchange gasses. In healthy individuals, RI values generally range from 0.8 to 1.2. An RI value exceeding this range may signal problems like inadequate oxygenation or carbon dioxide retention, which could lead to respiratory failure.

Data analysis demonstrated that the NRDS group had higher LUS, RI, and SOFA scores than the N-NRDS group, while OI values were lower in the NRDS group. This implies that more severe cases of NRDS at admission are associated with reduced OI values and worse prognoses. The results suggest that integrating LUS, OI, RI, and SOFA scores could effectively predict the severity of NRDS, potentially leading to improved treatment strategies and better clinical outcomes. The NRDS neonates were categorized into mild, moderate, and severe groups based on pulmonary disease severity. Results demonstrated that neonates with severe NRDS had significantly higher LUS, SOFA, and RI scores and the lowest OI values compared to those with mild and moderate NRDS, with scores of 27.12 ± 7.1, 111.7 ± 57.8, 19.36 ± 5.11, and 2.21 ± 1.12, respectively (*p* < 0.01).

The increasing incidence of neonatal pulmonary infections has made managing high-dimensional data with traditional statistical models increasingly tricky (29). In contrast, machine learning leverages large datasets to build highly efficient and precise mathematical models, reshaping traditional clinical practices and offering potentially optimal treatment solutions for patients. The integration of machine learning into clinical practice offers significant potential. For example, Khamzin et al. (31) investigated the use of machine learning for the intelligent analysis of CT and MRI images in medical imaging. Likewise, McCoubrey et al. (32) utilized machine learning to examine gut microbiome characteristics for evaluating drug metabolism. Currently, machine learning is also being used for sensitivity analysis in pneumonia. For example, Lu et al. (33) and Liu et al. (34) integrated machine learning with Raman spectroscopy and whole-genome sequencing to evaluate resistance levels in *Klebsiella pneumoniae*. Nevertheless, Raman spectroscopy and whole-genome sequencing still need to be standard practices in microbial identification.

The use of artificial intelligence methods, such as machine learning, in clinical practice remains in its early exploratory phase. Combining clinical expertise with ultrasound data shows promising potential as machine learning algorithms become more sophisticated and reliable. Various machine learning algorithms differ in characteristics such as fitting performance, algorithmic complexity, and the capacity to manage multi-feature data. This study aims to develop a low-cost, high-precision diagnostic system for NRDS to aid clinicians in making timely and accurate decisions. To achieve this, the study constructed four machine-learning models to predict the severity of NRDS, including LR, RF, NN, and SVM. Among these models, the RF model performed the best in the training set, achieving an AUC value of 0.894. The RF model outperformed logistic regression, neural networks, and support vector machines in terms of speed and accuracy with large datasets. Conversely, the minimum prediction accuracy of SVM is 0.766, compared to 0.771 for LR, 0.808 for RF, and 0.786 for NN. This performance discrepancy may be attributed to the small sample size, the limited number of variables, and the relatively homogeneous study population. Furthermore, the RF model offers several advantages, including fewer statistical assumptions, robust noise tolerance, reduced risk of overfitting, and minimal need for parameter tuning.

## Conclusion

This study created four machine-learning models by combining clinical indicators with imaging features. The results show that the RF model exhibited the highest performance and is particularly effective in predicting NRDS severity. Furthermore, analyzing variable weights across the four algorithms revealed key predictors, including LUS, OI, SOFA, and RI. Using machine learning models can make more accurate clinical decisions for NRDS patients.

## Data Availability

The raw data supporting the conclusions of this article will be made available by the authors, without undue reservation.
